# Animal‐ and Plant‐Source Protein‐Rich Foods Differ in Their Association With Maternal Micronutrient Status and Breast Milk Composition During Lactation

**DOI:** 10.1111/mcn.70253

**Published:** 2026-09-23

**Authors:** Sofa Rahmannia, Gina Arena, Kevin Murray, Aly Diana, Lisa Houghton, Rosalind Gibson, Siobhan Hickling

**Affiliations:** ^1^ School of Population and Global Health The University of Western Australia Crawley Western Australia Australia; ^2^ Faculty of Medicine Universitas Pasundan Bandung West Java Indonesia; ^3^ Medical School The University of Western Australia Nedlands Western Australia Australia; ^4^ The Kids Research Institute Nedlands Western Australia Australia; ^5^ Nutrition Working Group, Faculty of Medicine Universitas Padjadjaran Bandung West Java Indonesia; ^6^ Department of Public Health, Faculty of Medicine Universitas Padjadjaran Bandung West Java Indonesia; ^7^ Department of Human Nutrition University of Otago Dunedin Otago New Zealand

## Abstract

Micronutrient deficiencies in lactating women remain widespread and under‐addressed, compromising maternal health and breast milk quality. Although dietary guidelines promote protein‐rich foods, most do not distinguish between animal and plant sources within this food group, despite differences in nutrient profiles and bioavailability. This study examined associations between intake of animal‐source and plant‐source foods within the protein‐rich food group and maternal nutritional biomarkers in blood and breast milk. Nutrient contributions from each source and intake patterns across sociodemographic groups were also assessed. Data were drawn from 220 mother–infant pairs in urban and rural West Java, Indonesia. Maternal dietary intake was assessed over 3 non‐consecutive days using a combination of weighed food records and recall. At 5 months postpartum, maternal BMI, blood, and breast milk samples were collected to assess zinc, iron, vitamin A, and B12 status. Associations between dietary intake and maternal biomarkers were analysed using multiple linear regression, adjusting for relevant covariates. Total protein‐rich food intake was not significantly associated with maternal biomarkers. However, higher intake of animal‐source protein‐rich foods was positively associated with serum B12 and zinc, while higher intake of plant‐source protein‐rich foods was inversely associated with breast milk vitamin A. Macronutrients, particularly protein and fat, from animal‐based foods were more strongly associated with maternal micronutrient biomarkers than micronutrient intake alone. Protein‐rich food intake did not vary across sociodemographic groups, indicating feasibility for broad‐based interventions. These findings support a shift in dietary guidance for lactating women, from focusing solely on total quantity of protein‐rich foods to recognising nutritionally meaningful differences between animal‐source and plant‐source foods.

## Summary

- Total intake of protein‐rich foods was not associated with maternal micronutrient biomarkers during lactation.
- When disaggregated by source, animal‐based protein intake was positively associated with maternal serum vitamin B12 and zinc, while plant‐based protein intake was inversely associated with breast milk vitamin A.
- Macronutrients derived from animal‐based foods, particularly protein and fat, showed stronger associations with maternal micronutrient status than micronutrient intake alone.
- Protein‐rich food consumption did not differ across sociodemographic groups, suggesting feasibility for population‐level dietary guidance.
- Dietary guidelines for lactating women may benefit from distinguishing protein‐rich food sources rather than focusing solely on total intake of protein‐rich foods.

## Graphical abstract

Intake of animal‐source and plant‐source foods within the protein‐rich food group showed differential associations with maternal and breastmilk micronutrient biomarkers during lactation. Higher intake of animal‐source protein‐rich foods was positively associated with serum zinc and vitamin B12, whereas higher intake of plant‐source protein‐rich foods was inversely associated with breastmilk vitamin A in this cohort.

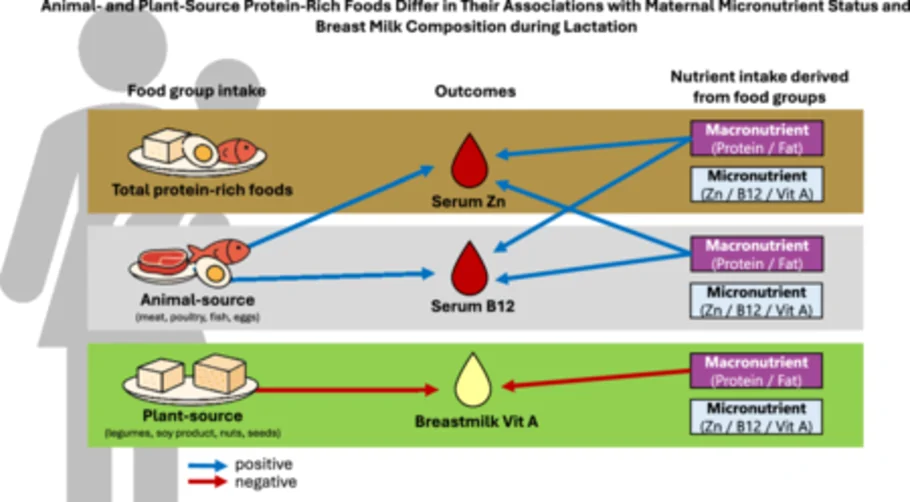

## 1 Introduction

Nutritional demands increase substantially during lactation to support both maternal health and breast milk production. Micronutrient deficiencies during this period have been linked to adverse maternal outcomes, compromised breast milk composition, and suboptimal infant growth and development (Moran 2017). Despite these risks, deficiencies in key micronutrients such as iron, zinc, vitamin A, and vitamin B12 remain highly prevalent among lactating women in low‐ and middle‐income countries (LMICs), including Indonesia (Gibson et al. 2020), and are also observed in higher‐income settings (Abe et al. 2016).

Various strategies have been implemented to address maternal micronutrient deficiencies, including supplementation and food fortification. However, the evidence for their effectiveness is mixed across populations, and concerns have been raised regarding their sustainability, accessibility, and long‐term impact (Berti et al. 2018; Bhutta et al. 2013; Victora et al. 2012). These approaches often operate as external interventions that rely on programme infrastructure and consistent distribution. In contrast, food‐based strategies are embedded in daily eating practices, making them more inherently accessible, acceptable, and sustainable. With appropriate awareness and supportive behaviours, food‐based approaches can serve as a more empowering and lasting solution to improve maternal nutrition (Kurian et al. 2021; Morón 2006).

One such approach involves translating nutritional needs into food‐based dietary guidelines. These guidelines are intended to guide optimal dietary practices, including during lactation. However, our previous analysis found that while adherence to the Indonesian dietary guidelines, particularly in consuming protein‐rich foods, was relatively high, it was not associated with improved maternal micronutrient biomarkers (Rahmannia et al. 2025). It is possible that current food‐based recommendations may be too broad and ambiguously interpreted to effectively influence nutritional status.

One possible explanation is that several dietary guidelines, including those from Indonesia, group diverse protein‐rich food sources such as meat, fish, eggs, and legumes under a single ‘protein‐rich food’ category (Rahmannia et al. 2025). They typically do not distinguish between animal‐ and plant‐based protein‐rich food sources, despite well‐established differences in nutrient composition and bioavailability (Ministry of Health Republic Indonesia 2014; National Health and Medical Research Council. 2013; Nutrition Division ‐ Ministry of Health 2011). Animal‐based foods provide highly bioavailable forms of iron, zinc, and vitamin B12, along with fats that enhance the absorption of fat‐soluble vitamins (Sheffield et al. 2024), while plant‐based proteins may contain inhibitors such as phytates and polyphenols that reduce mineral absorption (Ali et al. 2022). Although fermented products like tempeh may mitigate some of these limitations (Samtiya et al. 2020), their contribution to maternal micronutrient adequacy depends on the broader dietary pattern, including dietary planning, fortification, and supplementation.

This discrepancy raises a critical question: do animal‐ and plant‐source foods within the protein‐rich food group differ in their association with maternal nutritional status? To address this, the present study investigated the associations between protein‐rich food intake, categorised into animal‐source and plant‐source food groups, and maternal nutritional biomarkers. Outcomes included serum concentrations of ferritin (iron status), zinc, retinol‐binding protein (vitamin A status), and vitamin B12, as well as breast milk levels of these micronutrients. Maternal body mass index (BMI) was also assessed as an indicator of postpartum nutritional and metabolic status. Although not a primary outcome, BMI was included to explore potential effects of protein‐rich food sources on energy balance and weight status, given the differing macronutrient profiles of animal‐ and plant‐based foods. Secondary objectives were to (1) analyse the sociodemographic characteristics associated with protein‐rich food intake from animal and plant sources, and (2) examine the relationship between nutrients derived from these food sources and maternal nutritional biomarkers.

## 2 Methods

### 2.1 Study Design and Participant Characteristics

This study follows lactating mothers and their infants over the first 2 years of life. Participants were recruited from urban (Bandung, *n* = 113) and rural (Sumedang, *n* = 107) areas in West Java, Indonesia. Eligible participants included mothers who were actively breastfeeding at the time of recruitment, had delivered full‐term infants, and whose infants weighed at least 2500 grams at birth. Mothers with chronic illnesses or acute malnutrition were excluded.

### 2.2 Maternal Sociodemographic Characteristics

Maternal sociodemographic characteristics were collected at 2 months postpartum through an interviewer‐administered questionnaire. Additional information on household conditions, including asset ownership, was also gathered. A household wealth index was then constructed using principal component analysis based on asset data, following the methodology recommended by the Demographic and Health Surveys (Fry et al. 2014).

### 2.3 Dietary Assessment and Analysis

Dietary intake was assessed over 3 non‐consecutive days, including 2 weekdays and 1 weekend day. Urban participants self‐weighed their food intake over a 24‐h period using detailed guidelines, while rural participants' food intake was weighed by trained community cadres over a 12‐h daytime period, supplemented with self‐weighing and recall for nighttime intake. Protein‐rich food sources were then classified into animal‐based protein and plant‐based protein. In this manuscript, the term *protein‐rich foods* refers to food groups commonly classified as protein foods in food‐based dietary guidelines, rather than protein as an isolated nutrient exposure. These foods were categorised into animal‐source foods (e.g., meat, poultry, fish, eggs, organ meats) and plant‐source foods (e.g., soy products, legumes, nuts, and seeds). Daily intakes were calculated for each protein food source, and macronutrient and micronutrient contributions were estimated using tailored food composition tables. The contribution of fortified wheat flour to total thiamine, riboflavin, iron, zinc, and folic acid intake was considered in the calculations (Ministry of Health of the Republic of Indonesia 2003). As part of the dietary data cleaning process, the Goldberg method was routinely applied to assess the plausibility of energy intake (Tooze et al. 2012); however, no participants were excluded from this analysis based on this criterion.

### 2.4 Outcome Measurements

At 5 months postpartum, maternal anthropometric measurements, blood samples, and breast milk samples were collected. Body mass index (BMI) was calculated and classified using the Asia‐Pacific criteria, with overweight defined as BMI ≥ 23.0 kg/m^2^ (World Health Organization‐Western Pacific Region et al. 2000). Blood samples were drawn by trained phlebotomists under non‐fasting conditions, as fasting was not required for this community‐based study of lactating women. The time of blood collection and time since the last meal were recorded. Samples were processed to separate serum and stored at −20°C until analysis (Centre de toxicologie du Québec, 2021). Serum concentrations of ferritin, retinol‐binding protein (RBP), C‐reactive protein (CRP), and α‐1‐acid glycoprotein (AGP) were analysed using sandwich enzyme‐linked immunosorbent assay at VitMin Lab, Germany (Erhardt et al. 2004). Soluble transferrin receptor (sTfR) was also measured to complement ferritin as an additional indicator of tissue iron status. Serum vitamin B12 was measured using electrochemiluminescence immunoassay (Roche Diagnostics, GmbH), and serum zinc was assessed using inductively coupled plasma mass spectrometry (ICP‐MS; Agilent 7500) at the Centre for Trace Element Analysis, University of Otago, New Zealand. Inflammation‐adjusted values for ferritin, sTfR, RBP, and zinc were derived using the BRINDA regression correction method (Suchdev et al. 2016), and serum zinc concentrations were further adjusted for time of sample collection and fasting duration, consistent with published recommendations for non‐fasting field studies (Arsenault et al. 2011). Cut‐off values for deficiency were defined as adjusted ferritin < 15 μg/L (World Health Organization and World Health Organization 2011), serum zinc < 10.7 μmol/L (Brown et al. 2004), RBP < 1.2 μmol/L (Engle‐Stone et al. 2011), and vitamin B12 < 221 pmol/L (Allen et al. 2018).

Breast milk volume at 5 months was estimated using the deuterium oxide dose‐to‐mother technique (Gibson et al. 2020). For nutrient analysis, full breast milk samples were collected in the morning at the study visit. Mothers were asked to refrain from breastfeeding on one breast for at least 2 h prior to collection. After washing hands and the selected breast with distilled deionized water, milk was expressed manually or by breast pump until the breast was emptied to obtain both foremilk and hindmilk. Samples were collected into acid‐washed containers, aliquoted using trace‐element free techniques, and protected from direct light exposure by wrapping aliquot tubes in aluminium foil before storage at −80°C until laboratory analysis. Iron and zinc concentrations were measured using ICP‐MS at the University of Otago. Vitamin B12 was analysed using chemiluminescent immunoassay at the USDA Western Human Nutrition Research Centre (WHNRC), USA, and retinol along with provitamin A carotenoids were assessed using high‐performance liquid chromatography (HPLC) at the same facility. Full details of collection procedures and laboratory methods are reported elsewhere (Daniels et al. 2019).

### 2.5 Statistical Analysis

Descriptive analysis was conducted to summarise participant characteristics, with categorical variables reported as counts (*n*) and proportions (%). The prevalence of micronutrient deficiencies was also reported as proportions.

Associations between intake of protein‐rich foods and sociodemographic characteristics were examined using multiple linear regression models. Intake of protein‐rich foods (g/day) was treated as a continuous outcome variable, while sociodemographic characteristics were included as categorical predictor variables. The first category of each sociodemographic variable served as the reference group for comparisons. Beta coefficients (*β*) and 95% confidence intervals (CIs) were reported to quantify differences in intake of protein‐rich foods across sociodemographic groups.

**Figure 1:**
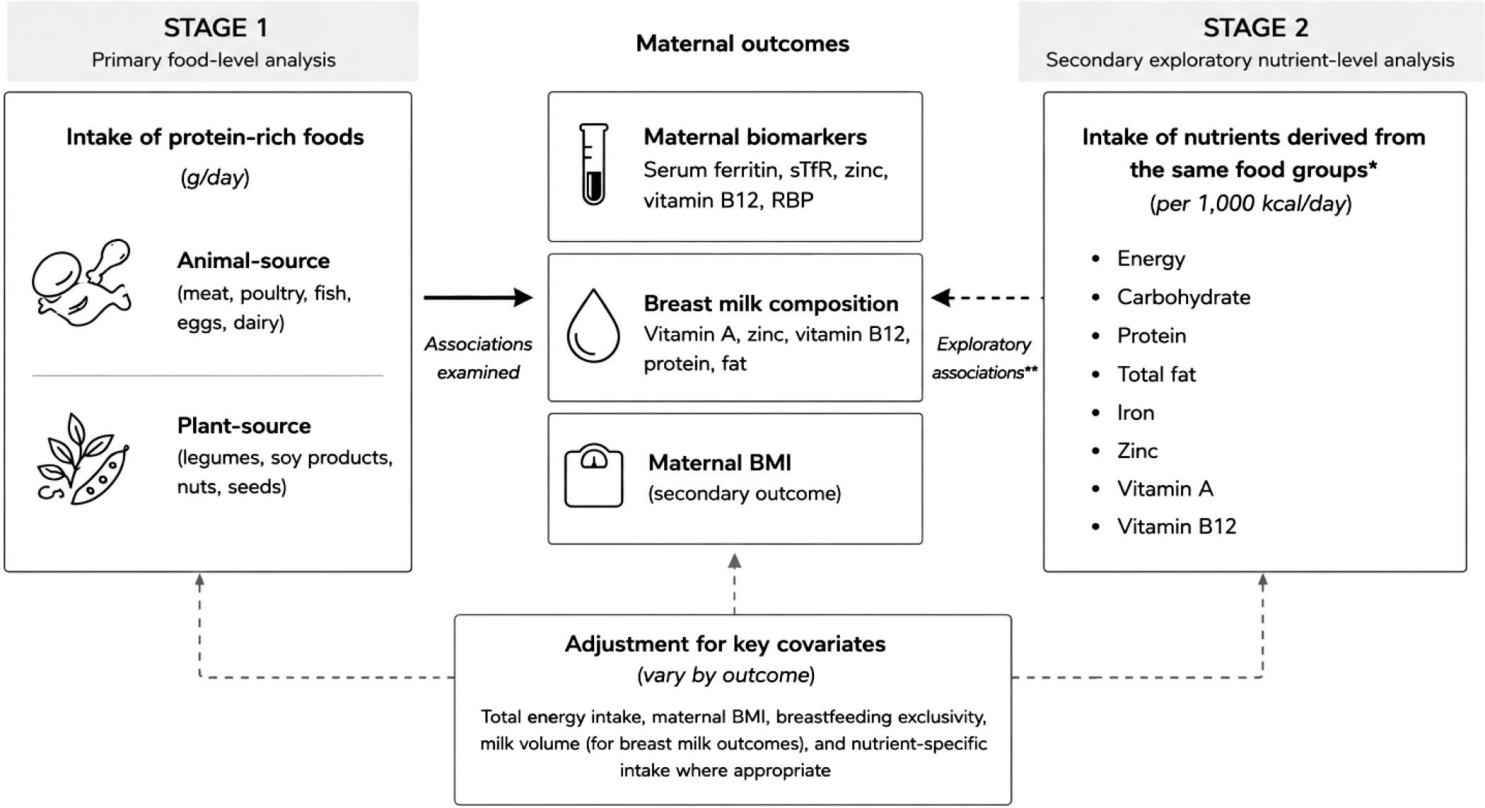
Conceptual framework of dietary analysis.

The diet‐biomarker analysis was conducted in two stages. In the first stage, the primary model examined associations between intake of protein‐rich foods, categorised into animal‐source and plant‐source food groups (g/day), and maternal nutritional outcomes. In the second stage, secondary exploratory nutrient‐level models examined nutrient intakes derived from those same food groups, including energy, macronutrients (carbohydrate, protein, fat), and selected micronutrients (e.g. iron, zinc, vitamin A, B12). These analyses were undertaken to assess whether specific nutrient components might help explain associations observed in the primary food‐level models. The conceptual framework of the dietary analysis is presented in Figure 1.

Primary outcomes included maternal blood biomarkers (serum zinc, ferritin, retinol‐binding protein (RBP), and serum B12), and breast milk composition (iron, zinc, vitamin A, and vitamin B12). Maternal BMI was also examined as a secondary anthropometric outcome.

Multiple linear regression models were used to estimate associations between dietary exposures and each outcome. The BMI models were adjusted for breastfeeding exclusivity and total energy intake. Models examining maternal blood biomarkers were adjusted for maternal BMI, total energy intake, breastfeeding exclusivity, and nutrient‐specific intake (e.g. iron intake for ferritin, zinc intake for serum zinc), except when the nutrient itself was the exposure variable. Models for breast milk composition were adjusted for milk volume, breastfeeding exclusivity, maternal energy intake, and nutrient‐specific intake. Covariates were selected a priori based on biological relevance and prior literature examining maternal nutritional biomarkers and breast milk composition during lactation. Adjustment variables were intended to account for factors potentially associated with both dietary intake and nutritional outcomes, including maternal nutritional status, lactation characteristics, overall dietary intake, and nutrient‐specific intake where appropriate. Results are presented as beta coefficients (β) with 95% confidence intervals (CIs). All statistical analyses were conducted using Stata (version 14.2), with statistical significance set at *p* < 0.05.

### 2.6 Ethical Statement

Ethical clearance for this study was granted by the Human Research Ethics Committee of The University of Western Australia (Ref: 2022/ET000721) and the Ethics Committee of the Faculty of Medicine, Universitas Padjadjaran, Indonesia (Ref: 05/UN6.C1.3.2/KEPK/PN/2017). Prior to participation, all individuals provided written informed consent. The study was conducted in line with the principles of the Declaration of Helsinki and complied with established ethical standards for research involving human subjects.

## 3 Results

The majority of participating mothers (74%) were within the optimal reproductive age range of 20–35 years (Table 1). Infant sex distribution was balanced, and participants were equally divided between urban (Bandung) and rural (Sumedang) areas. Educational attainment was generally low, with only 7% of mothers completing university education. Approximately half had attained only elementary (20%) or junior high school (35%) education. The majority (89%) were housewives, and 67% were multiparous. Household sizes were generally large, with more than half comprising more than four members.

**Table 1:** Sociodemographic and nutritional characteristics of participants.

Characteristics	*n*	%
Age		
< 20	23	10%
20–35	164	74%
> 35	33	16%
Infant sex		
Male	111	51%
Female	109	49%
Location		
Rural	107	49%
Urban	113	51%
Education		
Elementary school	43	20%
Junior high school	76	35%
Senior high school	85	39%
University	16	7%
Occupation		
Housewife	196	89%
Other	24	11%
Breastfeeding practice		
Exclusive breastfeeding at 2 months	182	83%
Exclusive breastfeeding at 5 months	91	43%
Parity		
Primiparous	72	33%
Multiparous	148	67%
Family size		
3–4	86	39%
5–6	80	36%
> 7	54	25%
Nutritional status		
Overweight and Obese	136	62%
Iron deficiency	44	25%
Zinc deficiency	101	59%
Vitamin A deficiency	60	34%
B12 deficiency	53	26%

*Note:* BMI was classified according to the Asia‐Pacific criteria, with overweight defined as BMI ≥ 23.0 kg/m^2^ (WHO, 2000). Nutritional deficiencies were determined using established biomarker thresholds. Iron deficiency was defined as serum ferritin < 12 μg/L (WHO, 2011), zinc deficiency as serum zinc < 9.9 µmol/L (Brown et al. 2004), vitamin A deficiency as retinol‐binding protein (RBP) < 0.83 μmol/L (Engle‐Stone et al. 2011), and vitamin B12 deficiency as serum B12 < 221 pmol/L (Allen et al. 2018). Ferritin, zinc, and RBP values were adjusted for inflammation using C‐reactive protein (CRP) and α‐1‐acid glycoprotein (AGP), following the BRINDA approach (Suchdev et al. 2016), serum zinc concentrations were also adjusted for diurnal variation and time since last meal (Arsenault et al. 2011).

Exclusive breastfeeding rates declined between 2 and 5 months postpartum. While 83% of mothers practised exclusive breastfeeding at 2 months, this figure dropped to 43% at 5 months. More than half (62%) of the participants were classified as overweight or obese. Micronutrient deficiencies were prevalent, with zinc deficiency affecting more than half (59%) of the participants, vitamin A deficiency present in one‐third (34%), and iron (25%) and vitamin B12 deficiencies (26%) observed in approximately one‐quarter of the population.

Protein‐rich food consumption patterns did not vary significantly across sociodemographic groups, including age, education level, economic status and family size (Table 2). Even between urban and rural areas, where differences in food accessibility might be expected, protein food intake from animal and plant sources remained similar. Among animal‐based proteins, beef (30%), chicken (26%) and eggs (26%) were consumed in nearly equal proportions, while fish (14%) and organ meats (4%) contributed less. Plant‐based protein intake was predominantly sourced from tempeh and tofu, both derived from soybeans, and accounted for 95% of plant‐source protein‐rich food consumption (data not shown).

**Table 2:** Sociodemographic characteristics and protein‐rich food intake: distribution and comparison by animal and plant sources.

Characteristics	*n*	Protein‐rich food intake (g/d)								
		Overall			Animal			Plant		
		Median (25, 75th)	Mean difference (95% CI)	*p*‐value	Median (25, 75th)	Mean difference (95% CI)	*p*‐value	Median (25, 75th)	Mean difference (95% CI)	*p*‐value
Age										
< 20	23	240 (192, 288)	ref		161 (127, 195)	ref		79 (47, 111)	ref	
20–35	164	241 (226, 257)	1 (−50, 52)	0.959	139 (129, 150)	−22 (−58, 14)	0.236	102 (92, 112)	23 (−11, 57)	0.183
> 35	33	224 (188, 260)	−16 (−80, 47)	0.613	130 (104, 155)	−31 (−76, 14)	0.170	94 (70, 118)	15 (−27, 57)	0.485
Location										
Rural	107	228 (207, 248)	ref		138 (123, 152)	ref		90 (76, 103)	ref	
Urban	113	249 (229, 269)	21 (−10, 52)	0.176	142 (128, 156)	5 (−17, 26)	0.685	106 (93, 120)	17 (−4, 38)	0.110
Education										
Elementary School	43	238 (205, 271)	ref		138 (114, 161)	ref		100 (78, 122)	ref	
Junior High School	76	241 (218, 265)	3 (−36, 43)	0.873	138 (121, 155)	0 (−28, 28)	0.984	103 (87, 119)	3 (−24, 29)	0.828
Senior High School	85	230 (207, 253)	−8 (−51, 35)	0.721	141 (124, 157)	3 (−28, 33)	0.854	90 (74, 105)	−11 (−40, 18)	0.466
University	16	269 (217, 322)	31 (−35, 97)	0.352	153 (116, 191)	16 (−31, 62)	0.512	116 (81, 151)	16 (−28, 60)	0.482
Occupation										
Housewife	196	238 (224, 252)	ref		138 (128, 148)	ref		100 (91, 109)	ref	
Other	24	244 (202, 286)	6 (−39, 51)	0.798	159 (130, 189)	21 (−10, 53)	0.183	84 (56, 112)	−16 (−45, 14)	0.304
Wealth index										
Lowest	44	220 (190, 251)	ref		130 (108, 151)	ref		91 (71, 111)	ref	
Second	46	259 (229, 289)	38 (−3, 80)	0.070	154 (133, 175)	25 (−5, 54)	0.100	105 (85, 125)	14 (−14, 42)	0.325
Middle	45	223 (194, 252)	2 (−40, 44)	0.912	133 (113, 154)	4 (−26, 34)	0.800	89 (70, 109)	−1 (−30, 27)	0.918
Fourth	44	245 (214, 276)	24 (−21, 69)	0.287	134 (112, 156)	4 (−27, 36)	0.785	111 (90, 132)	20 (−10, 50)	0.191
Highest	41	245 (212, 279)	25 (−23, 72)	0.305	150 (126, 174)	20 (−13, 54)	0.232	95 (73, 118)	4 (−27, 36)	0.787
Breastfeeding practice										
Partial breastfeeding	38	229 (196, 262)	ref		131 (107, 154)	ref		98 (76, 120)	ref	
Exclusive breastfeeding	182	240 (226, 255)	12 (−25, 48)	0.535	142 (132, 152)	11 (−15, 37)	0.388	98 (89, 108)	0 (−24, 25)	0.987
Parity										
Primiparous	72	257 (229, 284)	ref		156 (136, 175)	ref		101 (83, 119)	ref	
Multiparous	148	230 (212, 247)	−27 (−62, 9)	0.139	133 (120, 145)	−23 (−48, 2)	0.072	97 (85, 109)	−4 (−27, 20)	0.754
Family size										
3–4	86	242 (220, 264)	ref		136 (120, 152)	ref		106 (91, 121)	ref	
5–6	80	240 (218, 263)	−2 (−35, 31)	0.904	141 (125, 157)	5 (−18, 28)	0.684	99 (84, 114)	−7 (−29, 15)	0.541
> 7	54	230 (203, 257)	−12 (−47, 24)	0.521	146 (127, 165)	10 (−15, 35)	0.448	85 (67, 103)	−21 (−45, 2)	0.078

*Note:* “Ref” indicates the reference group for comparison in linear regression models. Differences in protein‐rich food intake (g/day) across sociodemographic groups were estimated using multivariable linear regression.

At first, we examined whether total protein‐rich food intake was associated with maternal nutritional biomarkers (Table 3). No significant associations were observed. However, when protein‐rich foods were disaggregated by source, distinct patterns emerged, particularly for animal‐based protein. This initial stage of analysis is presented in Table 3, while Table 4 builds on these findings by examining whether nutrient intake derived from different protein‐rich food sources helps explain the observed differences. Animal‐based protein intake showed consistent positive associations with key biomarkers, while plant‐based protein intake showed either none or was negatively associated. Specifically, a 100 g increase in animal‐based protein intake was associated with a rise in serum B12 by 25.7 pmol/L (95% CI: 2.6–48.7), whereas plant‐based protein intake showed no significant relationship. A similar pattern was observed for serum zinc: a 100 g increase in intake of animal‐source protein‐rich foods was linked to an increase of 0.3 μmol/L (95% CI: 0.1–0.5), with no effect observed for intake of plant‐source protein‐rich foods.

**Table 3:** Primary associations of animal‐source and plant‐source protein‐rich food intake with maternal biomarkers and breast milk composition (*n* = 220).

Outcomes	Median (25th, 75th)	Overall β (95% CI)	Animal‐source β (95% CI)	Plant‐source β (95% CI)
Maternal blood biomarkers				
Ferritin (μg/L)	27.3 (14.6, 50.7)	−2.4 (−6.4, 1.6)	−2.5 (−8.0, 3.0)	−2.2 (−7.9, 3.6)
Serum zinc (µmol/L)	10.3 (9.4, 11.2)	0.1 (−0.1, 0.3)	**0.3 (0.1, 0.5)**	−0.1 (−0.3, 0.1)
RBP (µmol/L)	1.3 (1.1, 1.6)	0 (−0.1, 0)	0 (0, 0.1)	−0.1 (−0.1, 0)
Serum vitamin B12 (pmol/L)	282 (218, 354)	11.9 (−4.3, 28.0)	**25.7 (2.6, 48.7)**	−1.1 (−23.5, 21.4)
Breastmilk composition				
Breast milk volume (mL/d)	718 (611, 817)	−1 (−27, 26)	1 (−37, 39)	−2 (−39, 35)
Breast milk iron (μg/L)	174 (126, 251)	14 (−14, 41)	26 (−12, 63)	0 (−39, 39)
Breast milk zinc (μg/L)	809.5 (563.5, 1175)	15 (−55, 85)	7 (−91, 104)	22 (−75, 119)
Breast milk vitamin A RAE (μg/L)	383 (271, 519)	−15 (−49, 19)	31 (−16, 79)	−**62 (−109, −15)**
Breast milk vitamin B12 (pmol/L)	203 (172, 253)	14 (−7, 36)	30 (−1, 61)	0 (−30, 30)
Secondary anthropometric outcome				
BMI (kg/m^2^)	23.8 (21.7, 26.8)	−0.4 (−0.9, 0.2)	−0.3 (−1.1, 0.5)	−0.4 (−1.2, 0.4)

*Note:* Beta coefficients (95% confidence intervals) represent the change in the outcome per 100 g/day increase in protein‐rich food intake, assessed separately for overall, animal‐source, and plant‐source food groups. BMI models were adjusted for breastfeeding exclusivity and total energy intake. Maternal biomarker models were adjusted for maternal BMI, breastfeeding exclusivity, total energy intake, and nutrient‐specific intake where appropriate. Breast milk composition models were adjusted for milk volume, breastfeeding exclusivity, maternal energy intake, and nutrient‐specific intake. Bolded values indicate statistically significant associations at *p* < 0.05.

**Table 4:** Secondary exploratory nutrient‐level associations with maternal biomarkers, breast milk composition, and BMI.

Outcome	Nutrient exposure	Overall β (95% CI)	Animal‐source β (95% CI)	Plant‐source β (95% CI)
Maternal blood biomarkers				
Ferritin (μg/L)	Median: 27.3 (14.6, 50.7)			
	Iron	−1.2 (−3.3, 0.9)	−0.6 (−3.6, 2.3)	−1.7 (−4.2, 0.9)
	Energy	−2.9 (−6.2, 0.3)	−3.1 (−6.9, 0.7)	−2.6 (−6.8, 1.7)
	Carbohydrate	−2.3 (−7.4, 2.8)	−2.0 (−9.7, 5.6)	−3.3 (−9.6, 3.0)
	Protein	−2.8 (−6.7, 1.1)	−3.3 (−7.4, 0.8)	−1.4 (−8.1, 5.3)
	Fat	−5.2 (−10.6, 0.2)	−5.9 (−12.1, 0.3)	−3.1 (−10.3, 4.0)
Serum zinc (µmol/L)	Median: 10.3 (9.4, 11.2)			
	Zinc	0.1 (0, 0.2)	0.1 (0, 0.2)	0 (−0.2, 0.2)
	Energy	**0.2 (0, 0.3)**	**0.3 (0.1, 0.4)**	0.1 (−0.1, 0.2)
	Carbohydrate	**0.2 (0, 0.4)**	**0.3 (0, 0.6)**	0.2 (−0.1, 0.5)
	Protein	**0.2 (0, 0.4)**	**0.2 (0, 0.4)**	0.1 (−0.2, 0.3)
	Fat	**0.3 (0, 0.5)**	**0.4 (0.1, 0.6)**	0.1 (−0.2, 0.4)
RBP (µmol/L)	Median: 1.3 (1.1, 1.6)			
	Vitamin A RAE	−0.1 (−0.2, 0.1)	−0.1 (−0.2, 0)	0.1 (−1.1, 1.2)
	Energy	0 (0, 0.1)	0 (0, 0.1)	0 (−0.1, 0)
	Carbohydrate	0 (−0.1, 0)	0 (−0.1, 0.1)	0 (−0.1, 0)
	Protein	0 (−0.1, 0)	0 (0, 0.1)	0 (−0.1, 0)
	Fat	0 (−0.1, 0.1)	0 (−0.1, 0.1)	0 (−0.1, 0.1)
Serum vitamin B12 (pmol/L)	Median: 282 (218, 354)			
	Vitamin B12	2 (−4.3, 8.4)	1.3 (−5.1, 7.6)	20 (−37.7, 77.8)
	Energy	9 (−2.7, 20.6)	**19.9 (5.2, 34.6)**	−5.4 (−20.4, 9.5)
	Carbohydrate	−1 (−18.9, 16.8)	3.6 (−22.4, 29.7)	−4.2 (−27.2, 18.7)
	Protein	**22 (7.5, 36.6)**	**27.9 (12.3, 43.5)**	−3.4 (−25.5, 18.8)
	Fat	**21.7 (2, 41.4)**	**34.8 (12, 57.6)**	−8.2 (−33.6, 17.2)
Breast milk composition				
Breast milk volume (mL/d)	Median: 718 (611, 817)			
	Energy	6 (−10, 21)	4 (−15, 24)	−3 (−25, 20)
	Carbohydrate	−18 (−43, 8)	−27 (−66, 11)	−18 (−52, 17)
	Protein	−5 (−25, 15)	−9 (−30, 13)	−9 (−42, 24)
	Fat	−10 (−38, 18)	−11 (−43, 22)	−22 (−60, 17)
Breast milk iron (μg/L)	Median: 174 (126, 251)			
	Iron	0 (−13, 13)	5 (−13, 23)	6 (−8, 21)
	Energy	0 (−20, 20)	4 (−19, 26)	5 (−24, 34)
	Carbohydrate	8 (−23, 39)	26 (−18, 69)	−9 (−54, 35)
	Protein	5 (−19, 28)	13 (−12, 38)	−12 (−54, 29)
	Fat	−8 (−40, 25)	−2 (−38, 35)	−7 (−55, 41)
Breast milk zinc (μg/L)	Median: 809.5 (563.5, 1175)			
	Zinc	3 (−41, 47)	−3 (−49, 42)	31 (−60, 123)
	Energy	41 (−10, 92)	27 (−35, 90)	29 (−41, 99)
	Carbohydrate	42 (−34, 117)	−29 (−139, 80)	98 (−10, 206)
	Protein	38 (−24, 100)	21 (−45, 88)	39 (−61, 138)
	Fat	75 (−8, 157)	55 (−39, 150)	55 (−63, 173)
Breast milk vitamin A RAE (μg/L)	Median: 383 (271, 519)			
	Vitamin A RAE	3 (−79, 86)	6 (−75, 88)	−437 (−1156, 282)
	Energy	2 (−20, 25)	17 (−10, 45)	−33 (−67, 2)
	Carbohydrate	15 (−23, 53)	25 (−30, 81)	−15 (−71, 40)
	Protein	7 (−22, 36)	24 (−8, 55)	−**55 (−105, −6)**
	Fat	−3 (−44, 38)	26 (−21, 74)	−**70 (−128, −11)**
Breast milk vitamin B12 (pmol/L)	Median: 203 (172, 253)			
	Vitamin B12	−2 (−11, 7)	−2 (−12, 7)	23 (−53, 98)
	Energy	6 (−10, 22)	12 (−9, 32)	7 (−15, 29)
	Carbohydrate	1 (−23, 26)	12 (−23, 47)	−7 (−42, 28)
	Protein	−1 (−21, 20)	6 (−16, 29)	−3 (−34, 29)
	Fat	13 (−14, 41)	19 (−13, 51)	8 (−30, 46)
Secondary anthropometric outcome				
BMI (kg/m^2^)	Median: 23.8 (21.7, 26.8)			
	Energy	−0.1 (−0.5, 0.2)	−0.2 (−0.6, 0.3)	−0.2 (−0.6, 0.3)
	Carbohydrate	−0.4 (−0.9, 0.2)	−0.1 (−1.0, 0.7)	−0.6 (−1.4, 0.1)
	Protein	−0.2 (−0.6, 0.2)	−0.2 (−0.7, 0.3)	−0.2 (−0.9, 0.5)
	Fat	−0.4 (−1.0, 0.3)	−0.4 (−1.2, 0.3)	−0.2 (−1.1, 0.6)

*Note:* Table 4 presents secondary exploratory nutrient‐level models conducted after the primary food‐level analyses shown in Table 3. These models examined whether specific nutrient components derived from animal‐source and plant‐source protein‐rich foods may help explain associations observed in the primary analyses. Beta coefficients (95% confidence intervals) represent the change in the outcome per 1 mg/day increase in micronutrient intake, 10 g/day increase in macronutrient intake, and 100 kcal/day increase in energy intake. Model adjustments followed the same approach as in Table 3. Bolded values indicate statistically significant associations at *p* < 0.05.

In contrast, intake of plant‐source protein‐rich foods was significantly and negatively associated with breast milk vitamin A. A 100 g increase in intake of plant‐source protein‐rich foods was associated with a 62 μg/L reduction (95% CI: −109 to −15). No significant associations were found between either protein‐rich food source and serum ferritin, breast milk zinc and iron, maternal BMI, or breast milk volume. Likewise, no associations were observed between intake of protein‐rich foods and sTfR, a functional indicator of iron status that reflects tissue iron status (data not shown).

To further explore these associations, we analysed macro‐ and micro‐nutrient intakes derived from protein‐rich food sources. Protein and fat intake from animal sources showed stronger associations with maternal nutritional biomarkers than micronutrient intake from these sources alone. A 10 g/day increase in protein from animal foods was associated with higher serum B12 (27.9 pmol/L, 95% CI: 12.3–43.5); a similar association was observed for animal fat intake (34.8 pmol/L, 95% CI: 12–57.6). Interestingly, when examining dietary vitamin B12 intake from all food sources overall no significant association with serum B12 levels was observed.

Similarly, serum zinc concentrations were positively associated with both protein (0.2 μmol/L per 10 g/day, 95% CI: 0–0.4) and fat intake (0.4 μmol/L per 10 g/day, 95% CI: 0.1–0.6) from animal‐source foods, whereas dietary zinc intake overall did not show a significant correlation.

Consistent with the earlier findings, plant‐derived protein and fat intakes were negatively associated with breast milk vitamin A. A 10 g/day increase in either was linked to a 55 μg/L decrease (95% CI: −105 to −6). No significant associations were found between dietary iron or any macronutrient intake and serum ferritin levels in lactating mothers.

## 4 Discussion

This study contributes to the growing evidence that the foods within the protein‐rich food group may differ in their associations with maternal biomarkers depending on whether they are animal‐source or plant‐source foods. While the total intake of protein‐rich food was not associated with serum or breast milk micronutrient biomarkers, disaggregating protein‐rich foods by source revealed contrasting relationships. These findings highlight the need to revisit current dietary guidance for lactating women, which often promotes protein‐rich food intake without distinguishing between animal and plant sources. In populations where micronutrient deficiencies remain prevalent, recognising such distinctions could improve the performance and impact of dietary recommendations.

### 4.1 Animal‐ and Plant‐Source Protein‐Rich Foods: Implications for Maternal Micronutrient Status

Disaggregated analysis revealed that intake of protein from animal sources was positively associated with serum zinc and B12, two critical micronutrients for maternal and infant health. The association between protein intake from animal‐source foods and serum zinc is consistent with evidence that zinc from animal foods is more bioavailable than zinc from plant sources, which often contain phytate, a strong inhibitor of zinc absorption (Allen 2008; Consalez et al. 2022; Grillenberger et al. 2006). Likewise, serum B12 levels were positively associated with protein intake from animal‐source foods, aligning with prior studies showing correlations between animal‐based food consumption and B12 status (Anaya‐Loyola et al. 2020). Vitamin B12, which is important for neurological development and hematopoietic function, is naturally concentrated in animal‐source foods; requirements during lactation may also be met through appropriately fortified foods or supplementation (Allen 2008; Grillenberger et al. 2006). Unlike vitamin B12 and zinc, serum ferritin, as an indicator of the level of iron stores, was not associated with protein intake from any source. This is not surprising given that the major factor influencing absorption and utilisation of iron is the iron status of the individual; dietary modifiers of iron absorption have only a modest effect (He et al. 2016; Herran et al. 2020). Furthermore, serum ferritin concentrations are also affected by factors other than the size of the iron stores, including the high prevalence of overweight and obesity in our sample. Excess adiposity is associated with chronic low‐grade inflammation, which elevates hepcidin, a hepatic hormone that downregulates intestinal iron absorption and inhibits iron release from stores, thereby impairing iron utilisation despite normal ferritin concentrations (Aguree and Reddy 2021). Although we adjusted ferritin for inflammation using CRP and AGP, this may not fully capture hepcidin‐related changes (Lim et al. 2016). sTfR was also examined, a marker of tissue‐level iron deficiency that is less influenced by inflammation. However, no association with protein intake was found, even after adjusting for BMI (Cepeda‐Lopez et al. 2019). One possible explanation is the influence of vitamin B12 status: low B12 can stimulate erythropoiesis, which increases sTfR levels (Gibson and Friel 2025). In our cohort, 26% of mothers were vitamin B12 deficient which may have contributed to elevated sTfR and obscured dietary associations. Only a few studies have reported positive associations between red meat consumption and improved ferritin (He et al. 2016; Herran et al. 2020). To our knowledge, such a relationship has not been explored in lactating women.

These findings should not be interpreted as discouraging plant‐based dietary patterns. Rather, they highlight that foods grouped within the broader protein‐rich food category may differ in micronutrient density and bioavailability, which may be relevant when considering nutritional needs during lactation. Plant‐source foods remain important contributors to dietary diversity and overall dietary quality.

The inverse association observed between plant‐based protein intake and breast milk vitamin A occurred despite no significant relationship with maternal serum retinol‐binding protein (RBP), a biomarker of vitamin A status. This divergence suggests that breast milk composition may be more sensitive to recent dietary intake and nutrient interactions than serum retinol biomarkers, which are tightly homeostatically controlled, remaining relatively constant, and do not reflect total body reserves of vitamin A unless liver vitamin A stores are severely depleted or excessively high (Fortuna et al. 2003). Several physiological mechanisms may explain this pattern. First, zinc plays a key role in both the synthesis of RBP and the enzymatic conversion of carotenoids to retinol. In this cohort, zinc deficiency was highly prevalent (59%), which may have impaired the bioconversion of provitamin A carotenoids to retinol, resulting in a low concentration of vitamin A in breast milk (Dijkhuizen et al. 2004).

Second, the bioavailability of vitamin A from plant‐based foods is inherently lower than from animal‐source foods, as the former provides provitamin A carotenoids rather than preformed retinol (Allen 2008). Efficient conversion of these carotenoids requires not only zinc but also adequate dietary fat, a nutrient that may be limited in predominantly plant‐based diets. Notably, tofu and tempeh, the primary plant protein‐rich food sources in this population, are low in fat, which is essential for carotenoid absorption (Yao et al. 2022). Taken together, these findings suggest that reduced intake of bioavailable vitamin A, along with low dietary fat, are additional factors constraining vitamin A transfer into breast milk.

The observed differences between animal‐ and plant‐based foods may reflect differences in food matrices, nutrient density, fat content, and micronutrient bioavailability. Animal‐based foods contain the so‐called “meat factor,” which facilitates the absorption of iron and possibly zinc from the diet (Consalez et al. 2022; Etcheverry et al. 2006). Conversely, plant‐source protein‐rich foods often contain anti‐nutritional factors such as phytates that hinder the absorption of some minerals. Although fermentation in plant foods like tempeh, which was commonly consumed in this population, can reduce phytate levels (Gibson et al. 2000), predominantly plant‐based diets may require particular attention to iron, zinc, and vitamin B12 adequacy during lactation through dietary planning, fortification, or supplementation (Allen 2008).

### 4.2 Implications for Dietary Guidelines and Food‐Based Interventions

Despite clear differences in nutrient density and bioavailability, most national dietary guidelines group animal and plant protein‐rich food sources under broad categories like “protein foods” or “meat, fish, legumes.” This overlooks the distinct nutritional roles of each. Our findings support considering each protein‐rich food source as potentially relevant to vitamin B12 and zinc guidance during lactation. Reviews of food‐based interventions have also concluded that food‐based interventions incorporating nutrient‐dense animal‐source foods have been associated with improved micronutrient outcomes (Allen 2008).

International dietary guidelines vary in whether animal‐source foods are treated as substitutable (Rahmannia et al. 2024). Our findings support source‐specific guidance during lactation, particularly for micronutrients with limited plant bioavailability (e.g., B12, zinc).

Encouragingly, this study found that protein‐rich food intake was consistent across all socioeconomic groups, with no notable differences by education, income, or urban–rural residence. This pattern persisted even when disaggregated by animal‐ and plant‐based sources, suggesting both types are culturally embedded and broadly accessible. Among animal‐source protein‐rich foods, beef, chicken, and eggs were most commonly consumed, while fish and organ meats were less common, consistent with prior reports (Rahmannia et al. 2019). Intake of plant‐source protein‐rich foods was dominated by soy‐based foods such as tofu and tempeh.

These patterns present an opportunity: widely consumed, nutrient‐dense protein foods that are already part of local diets offer a strong foundation for targeted nutrition guidance. Providing source‐specific guidance on nutrient density and bioavailability may be more feasible, acceptable, and equitable than promoting general increases in intake of protein‐rich foods.

### 4.3 Nutritional Considerations for Plant‐Based Dietary Patterns During Lactation

While this study shows stronger associations between animal‐based protein food intake and key maternal biomarkers (including serum B12 and zinc), it is important to acknowledge those women who consume little or no animal products due to personal, cultural, environmental, or religious reasons. These findings should not be interpreted as discouraging plant‐based dietary patterns. Rather, they suggest that foods grouped within the broader protein‐rich food category may differ in nutrient density and bioavailability, which may be relevant when considering nutritional needs during lactation. In this cohort, intake of plant‐source protein‐rich foods did not show the same biomarker associations as intake of animal‐source protein‐rich foods and was inversely associated with breast milk vitamin A. This finding does not establish the nutritional adequacy or inadequacy of a complete plant‐based diet and may reflect differences in food matrices, nutrient density, fat content, and bioavailability between protein‐rich food sources.

Even in plant‐forward countries such as the Netherlands, dietary guidance during pregnancy recognises the nutritional challenges of limiting animal products (Health Council of the Netherlands 2021). While the Dutch Dietary Guidelines 2015 recommend a more plant‐based and less animal‐based diet overall, the Health Council of the Netherlands notes that reducing animal product intake becomes more difficult during pregnancy due to the increased need for key nutrients such as calcium, iodine, iron, and vitamin B12, which are commonly supplied by animal‐based foods. The committee highlights that pregnant women who consume little or no animal products require extra attention to dietary quality and may need supplementation, especially for vitamin B12.

Global evidence supports this cautious approach. Nutrients like vitamin B12 and docosahexaenoic acid (DHA) tend to be lower in the breast milk of women following strict plant‐based diets unless supported by appropriate supplementation (Chrpová and Musilová 2023; Karcz and Królak‐Olejnik 2021). However, consistent access to fortified foods and supplements may not be guaranteed in many low‐ and middle‐income settings. At the same time, plant‐source foods remain important contributors to dietary diversity, affordability, and environmentally sustainable dietary patterns in many populations.

In Indonesia, current fortification policies cover micronutrients such as iron, zinc, vitamin B1, B2, and folic acid, but not vitamin B12, which is naturally supplied mainly by animal‐source foods (Ministry of Health of the Republic of Indonesia 2003). A recent modelling study of household‐level diets in Indonesia included some animal‐source foods in nutrient‐ and cost‐optimised dietary solutions designed to meet requirements for vitamin B12, iron, and zinc (de Pee et al. 2021). Moreover, predominantly plant‐based dietary models such as the EAT‐Lancet diet (Willett et al. 2019), while environmentally favourable, may require careful contextual adaptation to ensure nutritional adequacy for pregnant and lactating women in some low‐resource settings (de Pee et al. 2021). These tensions highlight the broader challenge of balancing micronutrient adequacy, sustainability goals, cultural dietary practices, and economic feasibility within dietary guidance for lactating women.

These findings point to a need for dietary guidance that is inclusive and accommodating of personal, religious, cultural, and environmental dietary pattern choices, yet evidence‐based. Ensuring adequate intake of bioavailable micronutrients remains important during lactation, whether achieved through animal‐source foods, fortified foods, supplementation, or carefully planned dietary strategies. At the same time, for women who avoid animal products, clearer guidance on intake of nutrient‐dense plant‐based foods, specific nutritional supplements and monitoring is essential. Nutrition policies must balance sustainability goals with biological realities, ensuring optimal health outcomes for both mothers and infants during this critical period of life.

### 4.4 Rethinking Micronutrient Strategies: The Role of Macronutrients and Food Source

Traditional strategies to prevent deficiencies in lactating women often focus on boosting micronutrient intake through iron‐rich foods or B12 supplements. However, this approach may overlook the important role of macronutrients, particularly protein and fat, in supporting nutrient metabolism, absorption, and transport. Our findings suggest macronutrient intakes, particularly protein and fat derived from animal‐source foods showed stronger statistical associations with several biomarkers than absolute micronutrient intake alone in these models.

One of the strongest associations observed was between intakes of fat and protein derived from animal‐source foods and serum vitamin B12, a relationship which was more pronounced than the association with total dietary B12 intake. This finding is consistent with evidence from Lebanon, where serum B12 depletion was more closely linked to low consumption of animal‐based foods than to total B12 intake (Hoteit et al. 2024). Other studies have shown that natural B12 from animal foods is more bioavailable and better correlated with serum status than synthetic supplements (Damayanti et al. 2018; Greibe et al. 2019), and experimental trials further demonstrate that B12 from animal foods leads to higher tissue accumulation than equivalent supplement doses (Greibe et al. 2018).

A similar pattern was observed for serum zinc, which was positively associated with intakes of fat and protein derived from animal‐source foods but not with total dietary zinc. This aligns with evidence that protein status influences zinc metabolism, both through increased physiological demand and by enhancing absorption (Katimba et al. 2023; Norii 2005). Features of animal‐source protein‐rich food matrices, including their amino acid profiles, may influence zinc transport and absorption (Li et al. 2022). Population‐level data from NHANES 2011–2014 and meta‐analysis also showed no consistent association between zinc intake and serum levels, suggesting that protein status and albumin‐bound zinc may explain some of the variation in biomarker response (Hennigar et al. 2018; King 2018; Moran et al. 2012).

These associations reported here are biologically plausible. Macronutrients influence the absorption, transport, and utilisation of micronutrients, with food matrices and macronutrient composition influencing enzyme systems and metabolic pathways, and fat intake facilitating the absorption of fat‐soluble vitamins (A, D, E, K). The fat content of animal foods may enhance vitamin A bioavailability, potentially explaining observed associations with breast milk vitamin A. In addition, protein intake modulates the bioavailability of minerals such as magnesium and iron (Iturbide‐Casas et al. 2019). These findings highlight the value of considering whole dietary patterns and nutrient sources, not just isolated nutrient targets. These findings should not be interpreted as indicating a causal effect of protein itself, but rather suggest that foods grouped within the broader protein‐rich food category may differ in micronutrient density and bioavailability. These findings suggest that considering overall food sources and macronutrient composition may complement conventional micronutrient‐focused strategies during lactation.

This also reflects a growing shift in the literature, which recognises that food‐source‐specific nutrient contributions remain under‐analysed in lactation research (Rahmannia et al. 2025). Integrating both macronutrient quality and nutrient bioavailability into dietary recommendations, rather than simply promoting “micronutrient‐rich” foods, may offer more effective strategies to support maternal health during lactation.

Although maternal BMI was not a primary outcome, it was analysed to assess potential unintended effects of protein‐rich diets. No significant associations were found between intake of protein‐rich foods and BMI; therefore, no association between animal‐source protein‐rich food intake and postpartum BMI was detected in this cohort. This may reflect complex physiological factors in the postpartum period, such as hormonal changes, energy demands from lactation, and unmeasured variables like physical activity. Further research is warranted.

### 4.5 Strengths and Limitations

This study provides an analytical contribution by assessing nutrient intake disaggregated by food source (animal vs. plant) in relation to maternal micronutrient status during lactation, a dimension that remains underexplored in the literature (Rahmannia et al. 2025). By including both urban and rural populations, the study enhances its real‐world relevance, particularly for LMIC contexts where dietary practices and food access can vary widely. The use of locally contextualised food composition data (reflecting Indonesian dietary patterns and including nutrients from mandatory fortification) adds further rigour to intake estimation and interpretation.

Several limitations should also be considered. As this study was observational and cross‐sectional, causal relationships cannot be inferred, and reverse causation remains possible, including the possibility that maternal nutritional status may influence dietary choices. Although inflammatory markers (CRP and AGP) were incorporated into biomarker adjustment procedures, residual confounding cannot be fully excluded. Unmeasured factors such as food preparation methods, broader dietary quality, and other contextual dietary practices may also have influenced the observed associations. In addition, although several associations reached statistical significance, effect sizes were generally modest and should be interpreted primarily at the population and dietary‐pattern level rather than as large individual‐level effects.

Breast milk samples were collected at a single morning time point to improve standardisation; however, breast milk composition may vary across the day, and a single sample may not fully capture within‐day variation. Generalisability may be limited. These findings may not fully apply to settings with markedly different food systems, dietary patterns, or availability of protein‐rich foods. Caution is therefore warranted in extrapolating results to high‐income countries or regions where the types, accessibility, and cultural integration of plant‐ and animal‐source foods differ substantially. Future research in more diverse populations and with broader dietary patterns is needed to validate and extend these findings across global settings.

## 5 Conclusion and Policy Recommendations

This study highlights the importance of distinguishing between animal‐ and plant‐source foods, which are often grouped as protein‐rich foods, when evaluating maternal nutritional status. While total protein‐rich food intake showed no significant association with micronutrient biomarkers, intake of animal‐source protein‐rich foods was positively associated with serum B12 and zinc, whereas higher intake of plant‐source protein‐rich foods was inversely associated with breast milk vitamin A in this cohort. These findings may reflect differences in nutrient density, food matrices, and micronutrient bioavailability between protein‐rich food groups rather than the effects of protein itself. Secondary exploratory nutrient‐level analyses further suggested that macronutrient components derived from animal‐source foods, particularly protein and fat, showed stronger associations with several biomarkers than micronutrient intake alone. The absence of association with serum ferritin may reflect physiological changes during lactation or the influence of other dietary components on iron metabolism. Notably, protein‐rich food intake was consistent across sociodemographic groups, indicating broad acceptability and feasibility for nutrition interventions.

Dietary guidelines for lactating women may benefit from moving beyond general recommendations for “protein‐rich foods” by considering distinctions between animal‐source and plant‐source foods within the protein‐rich food group, given their differing contributions to micronutrient bioavailability. In settings where vitamin B12 and zinc inadequacy are common, dietary guidance may identify culturally acceptable animal‐source foods as one practical source of these nutrients, alongside nutrient‐dense plant‐source foods and, where needed, fortified foods or supplementation. Such guidance should be adapted to dietary preferences, local availability, affordability, and sustainability.

## Author Contributions

S.R., S.H., G.A., and K.M. designed the study. S.R., K.M., G.A., and S.H. developed the methodology. S.R. conducted the formal analysis. S.R. and A.D. performed the investigation and data curation. A.D., R.S.G., and L.A.H. provided resources, with project administration led by A.D. Funding was acquired by R.S.G. and L.A.H. S.H., K.M., and G.A. supervised the study. S.R. wrote the original draft of the manuscript. R.S.G., L.A.H., A.D., G.A., K.M., and S.H. reviewed and edited the manuscript. All authors have read and approved the final manuscript.

## Conflicts of Interest

The authors declare no conflicts of interest.

## Data Availability

The data that support the findings of this study are available from the corresponding author upon reasonable request.
